# The Utility of T2-Weighted MRI Radiomics in the Prediction of Post-Exenteration Disease Recurrence: A Multi-Centre Externally Validated Study via the PelvEx Collaborative

**DOI:** 10.3390/cancers17183061

**Published:** 2025-09-19

**Authors:** PelvEx Collaborative

**Keywords:** radiomics, oncology, MRI, advanced rectal cancer, recurrence

## Abstract

**Introduction:** Recurrence after pelvic exenteration remains a significant concern in patients with locally advanced rectal cancer (LARC). Therefore, there is a need for improved non-invasive predictive tools to aid in patient selection. Radiomics, which extracts quantitative imaging features, may help identify patients at greater risk of recurrence. This study aimed to develop and validate a radiomics-based nomogram using pre-treatment MRI to predict postoperative recurrence risk in LARC. **Methods:** The largest multicenter retrospective radiomics analysis of 191 patients with pathologically confirmed LARC treated at fourteen centres (2016–2018) was performed. All patients received neoadjuvant chemoradiotherapy followed by curative-intent exenterative surgery. Manual tumour segmentation was performed on pre-treatment T2-weighted MRI. Feature selection employed LASSO regression with 5-fold cross-validation across 1000 bootstrap samples. The most frequently selected features were used to construct a logistic regression model via stepwise backward selection. Model performance was assessed using ROC analysis, calibration plots, decision curve analysis, and internal validation with 1000 bootstraps. A nomogram was generated to enable individualized recurrence risk estimation. **Results:** Postoperative recurrence occurred in 51% (*n* = 98) of cases. Five radiomic features reflecting tumour heterogeneity, morphology, and texture were included in the final model. In multivariable analysis, all selected features were significantly associated with recurrence, with odds ratios ranging from 0.63 to 1.64. The model achieved an optimism-adjusted AUC of 0.70, indicating fair discrimination. Calibration plots showed good agreement between predicted and observed recurrence probabilities. Decision curve analysis confirmed clinical utility across relevant thresholds. A clinically interpretable nomogram was developed based on the final model. **Conclusions:** A radiomics-based model using preoperative MRI can predict recurrence in LARC. The derived nomogram provides a practical tool for preoperative risk assessment. Prospective validation is necessary.

## Simple Summary

Pelvic exenteration is a major operation performed for patients with advanced rectal cancer, but the risk of cancer returning after surgery remains high. Accurately identifying which patients are most likely to benefit from this treatment is important for surgical planning and patient counselling. In this study, we analyzed MRI scans from 191 patients treated in fourteen hospitals worldwide. We used a computer-based method called radiomics to extract detailed information about tumour shape and texture that cannot be seen by the human eye. We combined these features into a mathematical model that predicts the chance of cancer returning after surgery. The model showed good accuracy and was converted into a user-friendly tool, called a nomogram, that doctors can apply before surgery. This approach may help improve patient selection and guide personalized treatment, but further prospective testing is needed before routine clinical use.

## 1. Introduction

The management of locally advanced and recurrent rectal cancer involves radical surgical procedures, such as pelvic exenteration [1]. While this approach offers the potential for a cure, it is associated with substantial morbidity and the risk of both locoregional and distant disease recurrence [2,3,4,5,6]. Identifying patients at higher risk of recurrence after exenteration is essential for patient selection, customizing postoperative management, and patient counselling [1,7].

In recent years, radiomics—the high-throughput extraction of quantitative features from medical images—has become a transformative tool in surgical oncology [8,9]. By using advanced computational techniques, radiomics allows for the analysis of imaging biomarkers that go beyond traditional radiological assessments, offering diagnostic and prognostic potential [8,9,10]. In cases of pelvic cancer, MRI-based radiomics has shown promise in characterizing tumour heterogeneity and nodal spread, thus improving the prediction of clinical outcomes, including disease recurrence [11,12,13].

Despite these advances, the use of MRI radiomics to predict post-exenteration recurrence remains underexplored, particularly in a multicentre setting where external validation is crucial for clinical relevance. To address this, our study aims to develop and externally validate a predictive nomogram that incorporates T2-weighted MRI radiomics features. Using data from the PelvEx Collaborative, a multi-institutional group comprising 14 centers across 12 countries, focused on outcomes for patients who have undergone exenterative surgery, we aim to create a robust and clinically useful model for recurrence risk stratification in this high-risk patient group [14].

## 2. Materials and Methods

### 2.1. Study Design and Participants

This multi-institutional retrospective study was conducted in accordance with the principles of the Declaration of Helsinki (1964) and received ethical approval from the St James Hospital/TUH Joint Research Ethics Committee. Due to its retrospective design, the requirement for written informed consent was waived.

Between January 2016 and December 2018, 191 consecutive patients with locally advanced rectal cancer were retrospectively enrolled from fourteen tertiary centers across countries (with 5-year follow-up data). Radiomic analysis was performed on pooled data from both centers, with internal validation conducted using 1000 bootstrap samples. Eligible patients met the following inclusion criteria.

#### 2.1.1. Inclusion Criteria

- Histologically confirmed locally advanced rectal adenocarcinoma (LARC);
- Radiological staging: T4 with or without nodal involvement (N+);
- Pre-treatment staging MRI of the rectum is available;
- Absence of metastatic disease (M0) at presentation;
- Underwent pelvic exenteration with curative intent.

#### 2.1.2. Exclusion Criteria

- Palliative resection;
- Insufficient follow-up (<3 years);
- Distant metastases (M1) at presentation;
- Recurrent rectal cancer.

### 2.2. Treatment and Follow-Up

All patients received neoadjuvant therapy followed by exenterative surgery, as determined by multidisciplinary team consensus and according to institutional protocol. Surgical specimens were evaluated according to institutional pathology protocols, including TNM staging in accordance with the American Joint Committee on Cancer (8th edition). Postoperative surveillance included clinical examination, endoscopy, CT of the thorax, abdomen, and pelvis (CT-TAP), and pelvic MRI at intervals specified by local guidelines.

Local recurrence was defined as a confirmed tumour recurrence at the site of primary resection, identified by endoscopy, interventional radiology, and/or MRI. Distant metastases were confirmed through imaging and/or biopsy.

### 2.3. Imaging Acquisition and Segmentation

MRI acquisition was performed as part of routine staging using various models (individual imaging parameters available upon request). Pre-treatment T2-weighted MRI scans were retrieved from institutional PACS systems. Tumour segmentation was performed manually by experienced radiologists using 3D Slicer (v5.4.0), ensuring inclusion of the entire tumour volume while excluding intraluminal contents. T2-weighted imaging (T2WI) was selected as the primary MRI sequence for this study due to its universal availability across participating centres and its established role as the cornerstone of rectal cancer staging. This choice enhances the reproducibility of radiomic feature extraction and supports the generalizability of the model across diverse clinical settings. High-resolution T2WI has been shown to provide excellent diagnostic accuracy for evaluating tumor invasion into the mesorectal fascia and adjacent organs, which are critical for surgical planning and prognosis in rectal cancer patients [15].

To ensure segmentation quality, 20% of cases were independently segmented by a second observer. Inter-observer agreement was assessed using the Dice similarity coefficient (DSC), with values exceeding 0.85. Any discrepancies were resolved by consensus.

### 2.4. Radiomics Feature Extraction

Radiomic features were extracted using the open-source PyRadiomics platform (v3.0.1). Images were first converted to nrrd format and resampled to an isotropic voxel size of 1 × 1 × 1 mm^3^, in accordance with the Image Biomarker Standardisation Initiative (IBSI) guidelines. Features were extracted from the segmented tumour volumes on both the original and wavelet-decomposed images. Wavelet decomposition was performed into eight directional components (LLL, LLH, LHL, LHH, HLL, HLH, HHL, HHH). The extracted features included first-order statistics, texture matrices (GLCM, GLRLM, GLSZM, GLDM), and shape descriptors. All features were Z-score normalized before analysis.

### 2.5. Clinical and Histopathological Variables

Additional clinical and pathological variables evaluated for association with disease recurrence included patient age, sex, clinical nodal stage (cN), tumour differentiation grade, and tumour distance from the anal verge.

Missing clinical data was handled using multiple imputation for variables included in the modelling pipeline. This approach preserved the sample size and reduced bias by estimating missing values based on the observed data distribution. Sensitivity analyses confirmed that model performance remained consistent when including or excluding imputed cases.

### 2.6. Model Development

Radiomic feature selection was performed based on a multi-step procedure previously established by our group [16,17,18]. The process began by generating 1000 bootstrap resamples from the original dataset, with replacement. In each resample, pairwise mean absolute correlations were computed, and one feature from any pair with a correlation exceeding a threshold of |r| >0.9 was removed to reduce multicollinearity. Subsequently, feature selection was performed using the least absolute shrinkage and selection operator (LASSO) embedded within a logistic regression framework, with 5-fold cross-validation applied to each bootstrap iteration. Ref. [19] Features were then ranked based on the frequency of their selection across all resamples. From this ranking, the top-most frequently selected features were retained. In cases where multiple versions of the same feature were present due to wavelet transformations, the version with the highest overall selection frequency was chosen. These features were used in stepwise backward logistic regression models fitted across the bootstrap samples. The most consistently selected subset of features was identified and used to construct the final radiomics model. Model coefficients were estimated on the full original dataset. To minimize overfitting, no more than five variables were included in the model, ensuring an event-per-variable (EPV) ratio greater than [20].

### 2.7. Model Evaluation

Model discrimination was quantified using the area under the receiver operating characteristic curve (AUC). Additional performance metrics, including positive predictive value (PPV) and negative predictive value (NPV), were calculated using the classification threshold defined by the Youden index.

Calibration performance, which assesses the agreement between predicted probabilities and observed outcomes, was evaluated visually using calibration plots, where the diagonal line (x = y) represents perfect calibration. To estimate the degree of optimism in model performance, internal validation was conducted using 1000 bootstrap iterations. Bootstrap-corrected AUC values and calibration slopes were derived by subtracting the estimated optimism from the original performance metrics, following TRIPOD guideline recommendations.

Clinical utility was assessed via decision curve analysis (DCA), which estimates the net benefit of prediction models across a range of decision thresholds. Comparisons were made against default strategies of “Treat All” and “Treat None,” with the model offering the highest net benefit at a given threshold considered optimal.

To support clinical interpretation and individual risk assessment, a nomogram was generated based on the final radiomics model. All statistical analyses were performed using R software (version 4.4.2; R Foundation for Statistical Computing, Vienna, Austria).

## 3. Results

### 3.1. Patient Characteristics

A total of 191 patients with locally advanced rectal cancer were included in the final analysis. The median age was 61 years (interquartile range [IQR]: 53–71), with 48% (*n* = 92) aged ≤60 years and 52% (*n* = 99) aged >60 years. The cohort comprised 77 females (40%) and 114 males (60%).

The median BMI was 25.9 kg/m^2^ (IQR: 22.3–29.0). ASA classification was available in 135 patients, with 17% (*n* = 32) classified as ASA I, 49% (*n* = 94) as ASA II, 17% (*n* = 32) as ASA III, and 0.5% (*n* = 1) as ASA IV. Preoperative carcinoembryonic antigen (CEA) levels were available for 134 patients, with a median of 4.7 ng/mL (IQR: 2.0–15.1); elevated CEA (≥5 ng/mL) was noted in 46% (*n* = 62) of cases.

Histological differentiation was classified as poor in 17% (*n* = 32), moderate in 62% (*n* = 118), and well-differentiated in 21% (*n* = 41) of patients. The predominant histology was adenocarcinoma, accounting for most cases, with subtypes such as mucinous adenocarcinoma and tubular adenocarcinoma less frequently observed. Regarding nodal status, 29% (*n* = 55) were staged as N0, 40% (*n* = 76) as N1, and 31% (*n* = 60) as N2/N3.

Tumour location was reported as lower rectum in 70 patients (37%), middle rectum in 50 patients (26%), and upper rectum in 29 patients (15%). The median tumour distance from the anal verge was 6 cm (IQR: 4–10), with an even distribution above and below this threshold (≤6 cm: 50%; >6 cm: 50%).

Mutation analysis was available for a subset of patients, with limited reporting. MMR/MSI intact status was confirmed in 12 patients (6%), and isolated KRAS and NRAS mutations were reported in a small minority of cases.

Postoperative recurrence occurred in 98 patients (51%) during follow-up (Table 1).

### 3.2. Radiomic Signature

Five radiomic features were selected as part of the final predictive signature for postoperative disease recurrence (Table 2):

- Wavelet LLL first-order Minimum (OR = 0.63; 95% CI: 0.44–0.90; *p* = 0.012).
- Wavelet LLL GLSZM Gray Level Non-Uniformity Normalized (OR = 1.57; 95% CI: 1.11–2.21; *p* = 0.011).
- Original shape Sphericity (OR = 1.54; 95% CI: 1.11–2.14; *p* = 0.010).
- Original GLCM Cluster Shade (OR = 0.66; 95% CI: 0.47–0.93; *p* = 0.016).
- Wavelet LLH GLCM IMC2 (OR = 1.64; 95% CI: 1.15–2.34; *p* = 0.006).

These features were significantly associated with recurrence status in multivariable logistic regression analysis. Notably, both shape- and texture-based parameters contributed to the radiomic model, suggesting that tumour heterogeneity and morphology are important predictors of recurrence. Individual definitions of Pyradiomics features can be found in Table 3.

**Table 2:** Multivariable logistic regression results of the final radiomic signature for predicting postoperative recurrence.

	OR	Lower CI	Upper CI	P
(Intercept)	1.045	0.769	1.419	0.779
Wavelet LLL first order Minimum	0.628	0.436	0.904	* 0.012
Wavelet LLL glszm Gray Level Non Uniformity Normalized	1.565	1.109	2.209	* 0.011
Original shape Sphericity	1.54	1.109	2.139	* 0.01
Original glcm Cluster Shade	0.659	0.469	0.926	* 0.016
Wavelet LLH glcm Imc2	1.64	1.152	2.335	* 0.006

*: Asterisk denotes statistically significant *p*-value.

**Table 3:** Definitions of PyRadiomics features [21,22,23].

Feature	Type	Definition
(Intercept)	Model coefficient	Baseline log-odds of recurrence when all feature values are zero.
Wavelet LLL First-Order Minimum	First-order (wavelet)	Minimum voxel intensity after applying low-pass filters in all directions (LLL). Reflects lowest signal region.
Wavelet LLL GLSZM Gray Level Non-Uniformity Normalized	Texture (GLSZM, wavelet)	Measures gray-level variability across homogeneous zones. Higher values = greater intensity heterogeneity.
Original Shape Sphericity	Shape	Quantifies how spherical the tumour is. Values closer to 1 = more spherical.
Original GLCM Cluster Shade	Texture (GLCM)	Reflects asymmetry in voxel intensity distribution. Higher values = more heterogeneity.
Wavelet LLH GLCM IMC2	Texture (GLCM, wavelet)	Measures complexity of intensity relationships. Higher values = greater texture complexity.

### 3.3. Model Performance

#### 3.3.1. Model Performance and Validation

**Figure 1:**
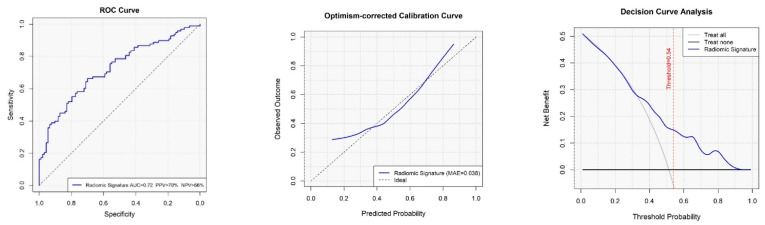
Discrimination performance of the radiomic model for predicting postoperative disease recurrence.

The final radiomic model achieved an optimism-corrected area under the receiver operating characteristic curve (AUC) of 0.70, indicating fair discriminatory performance (Figure 1). Calibration plots demonstrated good agreement between predicted and observed recurrence probabilities.

#### 3.3.2. Nomogram

**Figure 2:**
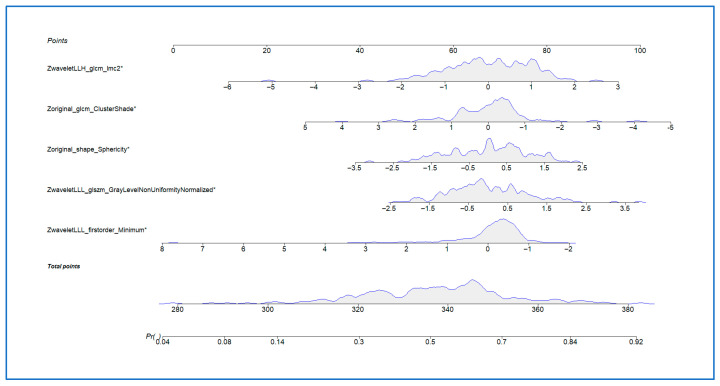
Nomogram based on the radiomic signature for individualized prediction of postoperative disease recurrence. *: Asterisks denotes statistically significant results.

A clinical nomogram was developed based on the final multivariate radiomic model to provide an individualized estimate of postoperative recurrence risk (Figure 2). This tool combines six selected radiomic features, each contributing a weighted score proportional to its predictive power. Each feature’s contribution is visualized along its own axis, allowing users to map individual patient values to corresponding point allocations. The cumulative score is then used to determine the overall recurrence risk probability, plotted on the lower axis.

In this cohort, the nomogram demonstrated good calibration, with predicted recurrence risks aligning with observed outcomes.

## 4. Discussion

This study represents the largest radiomics analysis in patients with LARC requiring pelvic exenteration. Unlike an earlier series, this study includes data from 191 patients treated across fourteen international centres [18]. This expanded multi-institutional dataset enabled the development of a robust MRI-based radiomics model and, importantly, its external validation. The ability to validate the model across different institutions ensures the generalizability and clinical usefulness of radiomics in rectal cancer prognosis.

Radiomics has demonstrated prognostic value in cancers such as prostate and bladder, where rigorous feature selection and cross-institutional validation informed robust model development. These methodological insights guided our approach, supporting the biological interpretability and clinical relevance of our MRI-based model in LARC [24,25].

Recurrence of disease after exenterative surgery in locally advanced rectal cancer remains a major clinical challenge. Despite employing neoadjuvant therapy and increasingly accepting radical surgery, recurrence rates are still high, as shown in our cohort where 51% experienced either local or distant disease progression. Traditional clinical and pathological factors like tumour grade, nodal status, and CRM involvement offer helpful information but lack the detail needed for truly personalized risk assessment [18,26].

Radiomics provides a promising approach by turning standard imaging into a rich source of quantitative data. In this study, we extracted high-dimensional radiomic features from routine pre-treatment T2-weighted MRI scans, enabling us to non-invasively examine tumour heterogeneity, shape, and textural complexity. Using a thoroughly validated selection and modelling process that included LASSO regression, bootstrapped internal validation, and feature consolidation, we developed a five-feature radiomic signature capable of predicting postoperative recurrence with reasonable accuracy. (optimism-corrected AUC = 0.70). The optimism-corrected AUC of 0.70 for our MRI-based radiomic model compares favourably with previously reported rectal cancer prognostic models, which have shown AUCs in the range of 0.60–0.68 using clinical and pathological factors alone. This improvement underscores the potential added value of quantitative imaging biomarkers for personalized risk assessment [27].

Including data from multiple institutions is a key strength, as it reduces the risk of overfitting to scanner-specific or protocol-dependent artifacts—a common criticism of radiomics studies. Refs. [28,29,30] By assessing how the model performs in a real-world, externally diverse population, this study offers the first evidence of external validity for a radiomics-based recurrence prediction model in LARC.

The selected features in the final model are biologically plausible and consistent with established tumour phenotypes [31]. Features such as *wavelet GLSZM Gray Level Non-Uniformity Normalized* and *GLCM IMC2* reflect tumour heterogeneity and spatial complexity-both of which are associated with aggressive phenotypes and poor outcomes [32]. Additionally, the inclusion of *shape sphericity*-a 3D morphological descriptor-suggests that tumour compactness may reflect underlying invasive potential. Prior studies in rectal and other pelvic malignancies have similarly identified shape descriptors as relevant prognostic markers [33]. The biological and clinical interpretability of these features enhances their translational potential. In line with advancements in imaging data augmentation, our study acknowledges the utility of Generative Adversarial Networks (GANs), such as the Pix2Pix model, which has been effectively employed in generating synthetic MRI datasets for brain tumor classification, thereby enhancing model robustness in scenarios with limited annotated data [19].

While these findings reinforce the biological significance of radiomic biomarkers, their validation in an externally diverse, surgically complex cohort supports the model’s potential generalizability [34,35,36]. The integration of radiomics-derived metrics into preoperative risk stratification frameworks could augment traditional staging, providing objective, non-invasive biomarkers to inform surgical planning, consideration of neoadjuvant therapy intensification, and postoperative surveillance.

To facilitate clinical adoption, we translated the radiomic model into a nomogram, providing an interpretable visual tool for individualized recurrence risk estimation [37,38]. Similarly to established nomograms used in breast and prostate cancer management (e.g., MSKCC nomogram), this tool could aid multidisciplinary decision-making in LARC, where choices regarding the extent of resection, adjunctive therapy, and follow-up intensity are still complex [39,40].

Nevertheless, limitations must be recognized. Although the model was validated externally, imaging acquisition protocols were not standardized across centres. While the model’s performance remained stable despite this variability—potentially indicating its robustness—prospective validation in a harmonized imaging environment is essential before clinical use. It should be noted that while our model was validated across multiple international centres, this validation was retrospective and constitutes cross-centre rather than truly independent prospective external validation; future prospective multi-institutional studies will be required to confirm the model’s generalisability in a real-world clinical setting. Additionally, relying solely on T2-weighted MRI limits feature diversity; adding multiparametric imaging (e.g., diffusion-weighted sequences) and molecular biomarkers could further improve predictive performance. Still, the model’s consistent performance across different institutions supports its robustness and potential for real-world clinical application.

## 5. Conclusions

This study demonstrates that an MRI-based radiomics model, built from standard preoperative imaging and validated across multiple institutions, can offer valuable predictions of postoperative recurrence risk in LARC. The creation of a nomogram improves its potential for use in routine clinical workflows and personalized cancer care.

## Figures and Tables

**Table 1:** Baseline demographic, clinical, and pathological characteristics of the study sample.

Characteristic	Total (N = 191)
**Age**	61 (53, 71)
≤60	92 (48%)
>60	99 (52%)
**Gender**	
Female	77 (40%)
Male	114 (60%)
**BMI (kg/m^2^)**	25.9 (22.3, 29.0)
**ASA classification (N = 135)**	
I	32 (17%)
II	94 (49%)
III	32 (17%)
IV	1 (0.5%)
**CEA (ng/mL) (N = 134)**	4.7 (2.0, 15.1)
CEA ≥ 5 ng/mL	62 (46%)
**Differentiation grade**	
Poor	32 (17%)
Moderate	118 (62%)
Well	41 (21%)
**cN stage**	
0	55 (29%)
1	76 (40%)
2–3	60 (31%)
**Distance from anal verge (cm)**	6 (4, 10)
≤6 cm	95 (50%)
>6 cm	96 (50%)
**Tumour location**	
Lower rectum	70 (37%)
Middle rectum	50 (26%)
Upper rectum	29 (15%)
Unspecified	6 (3%)
**Postoperative recurrence**	98 (51%)

Bold acts as headers for the below.

## Data Availability

The data are not publicly avaiable due to ethical restrictions.

## Appendix A

**Authors:** Niall J. O’Sullivan ^1,†^, Eimear T. Kyle ^1,†^, Juan Alvarado ^2^, Mirac Ajredini ^3^, Jose Manuel Asencio ^4^, Erman Aytac ^3^, Rumeysa Atabey ^3^, Funda Barlık ^5^, Sergei Bedrikovetski ^6^, Cecilia Besa ^7^, Nina Boc ^8^, Erik Brecelj ^8^, Aras Emre Canda ^9^, Alison Corr ^10^, Cagla Dinler ^5^, Ashley Duby ^11^, Tamara Glyn ^12^, Samantha K. Hendren ^13^, Jani Izlakar ^8^, Yukihide Kanemitsu ^14^, Silvia Kayser ^4^, Ales Kukovic ^8^, Jose T. Larach ^2^, Marjolein Liedenbaum ^15^, Berke Manoğlu ^5^, James F.M. Meaney ^10^, Paul H. McCormick ^1^, Isacco Montroni ^16^, Hiroshi Nagata ^14^, Leyla Ozer ^3^, Carlota Pérez-Carpio ^4^, Frank Pfeffer ^15^, Emauele Rausa ^16^, Scott E. Regenbogen ^13^, Malak Samaha ^11^, Tarik Sammour ^6^, Selman Sokmen ^5^, Bedriye Koyuncu Sokmen ^3^, Luca Sorrentino ^16^, Rutger Stijns ^17^, Giovanni Taffurelli ^16^, Yasuyuki Takamizawa ^14^, Hugo C. Temperley ^10^, Patricia Tejedor ^4^, Cem Terzi ^9^, Fariba Tohidinezhad ^18^, Shunsuke Tsukamoto ^14^, Greg Turner ^12^, Daniely Velayos ^4^, Raif Can Yarol ^5^, Ashish P. Wasnik ^19^, Johannes H.W. de Wilt ^17^, Desmond C. Winter ^20^, Michael E. Kelly ^1^.

**Affiliations:**

1 Trinity St. James Cancer Institute (TSJCI), Trinity College Dublin, St. James’s Hospital, Dublin, Ireland
2 Department of Digestive Surgery, Pontificia Universidad Católica De Chile, Santiago 8320165, Chile
3 Atakent Hospital Gastrointestinal Oncology Unit, Acibadem University, Istanbul 34638, Turkey
4 University Hospital Gregorio Marañón, 28007 Madrid, Spain
5 Diagnostic Radiology, Dokuz Eylul University Medical Faculty, Izmir 35220, Turkey
6 Colorectal Unit, Department of Surgery, Royal Adelaide Hospital, Adelaide 5000, Australia
7 Department of Radiology, Pontificia Universidad Católica De Chile, Santiago 8320165, Chile
8 Department of Radiology, Institute of Oncology, 1000 Ljubljana, Slovenia
9 KRC Clinic for Colorectal Surgery and Peritoneal Surface Malignancies, Izmir 35220, Turkey
10 Department of Radiology, St James’s Hospital, Dublin, Ireland
11 Faculty of Medicine, University of Michigan, Ann Arbor, MI 48109, USA
12 Christchurch Hospital, Christchurch 4710, New Zealand
13 Division of Colorectal Surgery, University of Michigan, Ann Arbor, MI 48109, USA
14 National Cancer Center, 5-1-1 Tsukiji, Chuo-Ku, Tokyo 104-0045, Japan
15 Department of Radiology, Haukeland University Hospital, 5009 Bergen, Norway
16 Colorectal Surgery Unit, Fondazione IRCCS Istituto Nazionale Dei Tumori, 20133 Milan, Italy
17 Surgery Department, Radboud University Medical Center, 6525 Nijmegen, The Netherlands
18 Department of Radiation Oncology (Maastro Clinic), School for Oncology and Reproduction (GROW), Maastricht University Medical Centre, 6229 Maastricht, The Netherlands
19 Division of Abdominal Radiology, Department of Radiology, University of Michigan, Ann Arbor, MI 48109, USA
20 Department of Surgery, St. Vincent’s University Hospital, Dublin, Ireland
† These authors contributed equally to this manuscript.

## References

[1] PelvEx Collaborative (2022). Contemporary Management of Locally Advanced and Recurrent Rectal Cancer: Views from the PelvEx Collaborative. Cancers.

[2] Ferenschild F.T., Vermaas M., Verhoef C., Ansink A.C., Kirkels W.J., Eggermont A.M.M., de Wilt J.H.W. (2009). Total pelvic exenteration for primary and recurrent malignancies. World J. Surg..

[3] PelvEx Collaborative (2024). Contemporary results from the PelvEx collaborative: Improvements in surgical outcomes for locally advanced and recurrent rectal cancer. Color. Dis..

[4] Brown K.G., Solomon M.J., Koh C.E., Sutton P.A., Aguiar S., Bezerra T.S., Clouston H.W., Desouza A., Dozois E.J., Ersryd A.L. (2024). Defining Benchmarks for Pelvic Exenteration Surgery: A Multicentre Analysis of Patients with Locally Advanced and Recurrent Rectal Cancer. Ann. Surg..

[5] Yang T.X., Morris D.L., Chua T.C. (2013). Pelvic Exenteration for Rectal Cancer: A Systematic Review. Dis. Colon Rectum.

[6] Shine R.J., Glyn T., Frizelle F. (2022). Pelvic exenteration: A review of current issues/controversies. ANZ J. Surg..

[7] Brown K.G., Morkaya J., Solomon M.J., Ng K.-S., White K., Sutton P., Winter D.C., Ansari N., Steffens D. (2024). Priority outcomes of pelvic exenteration for rectal cancer: A patient, carer, and clinician consensus. Br. J. Surg..

[8] Gillies R.J., Kinahan P.E., Hricak H. (2016). Radiomics: Images are more than pictures, they are data. Radiology.

[9] Parekh V., Jacobs M.A. (2016). Radiomics: A new application from established techniques. Expert Rev. Precis. Med. Drug Dev..

[10] Lambin P., Leijenaar R.T.H., Deist T.M., Peerlings J., de Jong E.E.C., van Timmeren J., Sanduleanu S., Larue R.T.H.M., Even A.J.G., Jochems A. (2017). Radiomics: The bridge between medical imaging and personalized medicine. Nat. Rev. Clin. Oncol..

[11] Li H., Chai L., Pu H., Yin L.-L., Li M., Zhang X., Liu Y.-S., Pang M.-H., Lu T. (2024). T2WI-based MRI radiomics for the prediction of preoperative extranodal extension and prognosis in resectable rectal cancer. Insights Imaging.

[12] Meng Y., Ai Q., Hu Y., Han H., Song C., Yuan G., Hou X., Weng W. (2024). Clinical development of MRI-based multi-sequence multi-regional radiomics model to predict lymph node metastasis in rectal cancer. Abdom. Radiol..

[13] Ma X., Shen F., Jia Y., Xia Y., Li Q., Lu J. (2019). MRI-based radiomics of rectal cancer: Preoperative assessment of the pathological features. BMC Med. Imaging.

[14] (2024). PelvEx Collaborative. https://www.pelvex.org/.

[15] Horvat N., Carlos Tavares Rocha C., Clemente Oliveira B., Petkovska I., Gollub M.J. (2019). MRI of Rectal Cancer: Tumor Staging, Imaging Techniques, and Management. Radiographics.

[16] Zhang Z., Wang Z., Yan M., Yu J., Dekker A., Zhao L., Wee L. (2023). Radiomics and dosiomics signature from whole lung predicts radiation pneumonitis: A model development study with prospective external validation and decision-curve analysis. Int. J. Radiat. Oncol. Biol. Phys..

[17] Compter I., Verduin M., Shi Z., Woodruff H.C., Smeenk R.J., Rozema T., Leijenaar R.T., Monshouwer R., Eekers D.B., Hoeben A. (2021). Deciphering the glioblastoma phenotype by computed tomography radiomics. Radiother. Oncol..

[18] O’Sullivan N.J., Tohidinezhad F., Temperley H.C., Ajredini M., Sokmen B.K., Atabey R., Ozer L., Aytac E., Corr A., Traverso A. (2025). Multi-Institutional MR-Derived Radiomics to Predict Post-Exenteration Disease Recurrence in Patients With T4 Rectal Cancer. Cancer Med..

[19] Onakpojeruo E.P., Sancar N. (2024). A Two-Stage Feature Selection Approach Based on Artificial Bee Colony and Adaptive LASSO in High-Dimensional Data. AppliedMath.

[20] Vittinghoff E., McCulloch C.E. (2007). Relaxing the rule of ten events per variable in logistic and Cox regression. Am. J. Epidemiol..

[21] Van Griethuysen J.J., Fedorov A., Parmar C., Hosny A., Aucoin N., Narayan V., Beets-Tan R.G.H., Fillion-Robin J.-C., Pieper S., Aerts H.J.W.L. (2017). Computational radiomics system to decode the radiographic phenotype. Cancer Res..

[22] Aerts H.J., Velazquez E.R., Leijenaar R.T., Parmar C., Grossmann P., Carvalho S., Bussink J., Monshouwer R., Haibe-Kains B., Rietveld D. (2014). Decoding tumour phenotype by noninvasive imaging using a quantitative radiomics approach. Nat. Commun..

[23] Zwanenburg A., Leger S., Vallières M., Löck S. (2016). Image biomarker standardisation initiative. arXiv.

[24] Huynh L.M., Bonebrake B., Tran J., Marasco J.T., Ahlering T.E., Wang S., Baine M.J. (2023). Multi-Institutional Development and Validation of a Radiomic Model to Predict Prostate Cancer Recurrence Following Radical Prostatectomy. J. Clin. Med..

[25] Zhang X., Xu X., Tian Q., Li B., Wu Y., Yang Z., Liang Z., Liu Y., Cui G., Lu H. (2017). Radiomics assessment of bladder cancer grade using texture features from diffusion-weighted imaging. J. Magn. Reson. Imaging.

[26] Chen K., Collins G., Wang H., Toh J.W.T. (2021). Pathological features and prognostication in colorectal cancer. Curr. Oncol..

[27] Kos F.T., Cecen Kaynak S., Akturk Esen S., Arslan H., Uncu D. (2024). Comparison of Different Machine Learning Models for Predicting Long-Term Overall Survival in Non-metastatic Colorectal Cancers. Cureus.

[28] Santini D., Danti G., Bicci E., Galluzzo A., Bettarini S., Busoni S., Innocenti T., Galli A., Miele V. (2023). Radiomic features are predictive of response in rectal cancer undergoing therapy. Diagnostics.

[29] Mayerhoefer M.E., Materka A., Langs G., Häggström I., Szczypiński P., Gibbs P., Cook G. (2020). Introduction to radiomics. J. Nucl. Med..

[30] Inchingolo R., Maino C., Cannella R., Vernuccio F., Cortese F., Dezio M., Pisani A.R., Giandola T., Gatti M., Giannini V. (2023). Radiomics in colorectal cancer patients. World J. Gastroenterol..

[31] Sanduleanu S., Woodruff H.C., De Jong E.E., van Timmeren J.E., Jochems A., Dubois L., Lambin P. (2018). Tracking tumor biology with radiomics: A systematic review utilizing a radiomics quality score. Radiother. Oncol..

[32] Sala E., Mema E., Himoto Y., Veeraraghavan H., Brenton J., Snyder A., Weigelt B., Vargas H. (2017). Unravelling tumour heterogeneity using next-generation imaging: Radiomics, radiogenomics, and habitat imaging. Clin. Radiol..

[33] Alvarez-Jimenez C., Antunes J.T., Talasila N., Bera K., Brady J.T., Gollamudi J., Marderstein E., Kalady M.F., Purysko A., Willis J.E. (2020). Radiomic texture and shape descriptors of the rectal environment on post-chemoradiation T2-weighted MRI are associated with pathologic tumor stage regression in rectal cancers: A retrospective, multi-institution study. Cancers.

[34] McQuerry J.A., Chang J.T., Bowtell D.D., Cohen A., Bild A.H. (2017). Mechanisms and clinical implications of tumor heterogeneity and convergence on recurrent phenotypes. J. Mol. Med..

[35] Brancato V., Garbino N., Aiello M., Salvatore M., Cavaliere C. (2024). Exploratory Analysis of Radiomics and Pathomics in Uterine Corpus Endometrial Carcinoma. Sci. Rep..

[36] García-Figueiras R., Baleato-González S., Padhani A.R., Luna-Alcalá A., Vallejo-Casas J.A., Sala E., Vilanova J.C., Koh D.-M., Herranz-Carnero M., Vargas H.A. (2019). How clinical imaging can assess cancer biology. Insights Imaging.

[37] Iasonos A., Schrag D., Raj G.V., Panageas K.S. (2008). How to build and interpret a nomogram for cancer prognosis. J. Clin. Oncol..

[38] Balachandran V.P., Gonen M., Smith J.J., DeMatteo R.P. (2015). Nomograms in oncology: More than meets the eye. Lancet Oncol..

[39] Rudloff U., Jacks L.M., Goldberg J.I., Wynveen C.A., Brogi E., Patil S., Van Zee K.J. (2010). Nomogram for predicting the risk of local recurrence after breast-conserving surgery for ductal carcinoma in situ. J. Clin. Oncol..

[40] Venclovas Z., Muilwijk T., Matjosaitis A.J., Jievaltas M., Joniau S., Milonas D. (2021). Head-to-head comparison of two nomograms predicting probability of lymph node invasion in prostate cancer and the therapeutic impact of higher nomogram threshold. J. Clin. Med..

